# Dysregulation of inhibitory control in young adult binge drinkers: Neuroimaging evidence of gender differences associated with misusing alcohol to cope with negative affect

**DOI:** 10.1016/j.addicn.2025.100243

**Published:** 2025-12

**Authors:** Austin B. Alderson Myers, David R. White, Ksenija Marinkovic

**Affiliations:** Department of Psychology, San Diego State University, 5500 Campanile Dr., San Diego, CA 92182, USA

**Keywords:** Binge alcohol, fMRI, Inhibitory control, Go/NoGo task, Drinking motives, Gender differences, Negative emotionality

## Abstract

Impaired inhibitory control contributes to compulsive drinking and increased risk of alcohol misuse. While the neural underpinnings of inhibitory control have been well documented, imaging evidence in binge drinkers (BDs) is scarce. Notably lacking are studies that consider gender differences, even though women are at greater risk of alcohol use disorder, cognitive deficits, and emotion dysregulation comorbidities. To address these gaps, the present study recruited young adult women and men (*N* = 83; women = 44) differing in levels of alcohol consumption who were scanned with fMRI during an inhibitory Go/NoGo task. Deficient inhibition in BDs was reflected in lower accuracy on NoGo trials and a tendency to respond faster than light drinkers (LDs) who drink regularly but in low-risk patterns. fMRI revealed greater activation to NoGo trials in BDs than LDs in the left inferolateral and medial frontal cortices and bilateral basal ganglia, which was positively associated with a recent history of binge drinking. Importantly, these differences were particularly pronounced in BD women, who showed enhanced activation in the left inferior frontal cortex (LIFC) compared to BD men. LIFC activation correlated with binge drinking and drinking to cope with negative emotions only in BD women. Moreover, greater LIFC activation mediated the impact of coping motives on binge drinking in BD women, suggesting that coping-related negative affect may exacerbate their alcohol misuse. This novel finding offers insight into potential gender differences in inhibitory control dysregulation in young BDs supporting other evidence of women’s heightened sensitivity to the effects of alcohol.

## 1. Introduction

Young adults frequently engage in binge drinking, which is characterized by intermittent bouts of heavy alcohol intake followed by periods of low use or abstinence. A binge episode is commonly defined as consuming 5+/4+ (men/women) standard alcoholic beverages in two hours, typically raising BAC to 0.08 % or above [1], but this benchmark is often exceeded [2–5]. Indeed, binge drinking has been associated with a greater risk of developing AUD and a tendency to engage in unsafe behaviors [6] along with cognitive and emotional impairments [7,8], anxiety and depression [9], cardiovascular and metabolic consequences [10,11], and cancer [12] amongst others. This is concerning given the high prevalence rates of binge drinking among young emerging adults [13], which may interfere with brain maturation [14]. Young adulthood is considered a time of personal discovery and experimentation with substances [15], and this form of alcohol use is commonly perceived to be socially acceptable and non-problematic [16–18]. Consequently, denial of alcohol-related problems is pervasive, and the inability to regulate alcohol intake may instead be perceived as normative development [19]. However, binge drinking is a potential pathway toward alcohol use disorder (AUD), which is characterized by the transition from impulsive to compulsive abuse of alcohol [20–22]. This is supported by longitudinal studies indicating that binge drinking during emerging adulthood predicts the development of AUD [23,24].

It has been established that acute alcohol intoxication acts principally upon the prefrontal networks underlying compromised top-down regulatory functions [25–32]. Furthermore, dysregulation of primarily prefrontal networks underlying inhibitory deficiencies is thought to play an essential role in AUD development [33–40]. Executive function has been proposed as one of the three major neurofunctional domains within the Addictions Neuroclinical Assessment (ANA), a neuroscience-based heuristic framework that can inform diagnosis and treatment [36,41]. Impaired inhibitory control is associated with binge drinking as it contributes to compulsive consumption and predicts alcohol misuse [42–44].

Inhibitory control refers to the ability to suppress behaviors considered undesirable within a specific situational context while enacting goal-specific actions [45,46]. It is commonly probed with the Go/NoGo task that relies on rapidly presenting frequent Go trials to establish a strong response prepotency that renders response withholding during rare inhibitory NoGo trials challenging [47]. Neuroimaging evidence indicates that inhibitory control elicits the blood-oxygen-level-dependent (BOLD) signal in distributed, primarily prefrontal regions such as the supplementary motor area (SMA) and bilateral inferior frontal cortices, along with temporal, parietal, and subcortical areas [45,48–51]. It has been suggested that the right inferior frontal cortex (IFC) is a principal region subserving inhibition processes [45,52] with additional contributions from the left IFC [53]. Inhibitory control engages attentional and working memory functions in addition to response inhibition [54,55]. A lot of the extant imaging evidence on the impact of binge drinking on inhibitory control indicates elevated prefrontal activation during successful response suppression in binge drinkers (BDs) relative to light drinkers (LDs) [56,57]. Other studies have reported no group differences [58] or weaker BOLD responses [59]. Greater BOLD activation in BDs has also been observed in studies probing cognitive control [60,61] and working memory [62]. These findings have been interpreted as a compensatory neural response to satisfy task demands, as demonstrated in studies of people with AUD [63]. However, this evidence has been obtained in small-scale studies with limited statistical power, so replication in larger samples is needed.

Furthermore, neuroimaging evidence on gender differences is lacking, even though women are at greater risk of AUD, cognitive deficits, and alcohol-related health harms despite lower alcohol intake than men [64–67]. While men have historically been drinking more heavily than women [68], this gap has been narrowing as women’s alcohol consumption and AUD rates approach those of men [69–71]. This trend is especially concerning, given women’s greater susceptibility to the effects of alcohol and their faster progression toward developing AUD [70, 72–74]. The evidence on gender differences in inhibitory control is mixed [75]. However, inhibitory deficits predict increased drinking levels after a 6-month delay among women college students [76], while impaired inhibition has been reported for heavy-drinking women [77]. Relatedly, women with AUD have higher rates of comorbid mood and anxiety disorders [78,79]. They are more likely to drink to cope with negative emotional states based on alcohol’s anxiolytic effects [70, 72–74,77,79]. Complementing executive deficits, negative emotionality is another essential neurofunctional domain within the ANA framework [36], which is associated with increased levels of alcohol consumption and relapse [80–82]. Drinking to alleviate negative experiences (i.e., coping motive) mediates the predictive impact of negative emotionality on alcohol consumption in individuals receiving AUD treatment across a year-long follow-up [83]. Given the scarcity of extant evidence on the early stages of alcohol misuse, research is needed to examine the neural underpinnings of gender differences in inhibitory control and the impact of coping motives on alcohol intake in young adult binge drinkers.

To address these gaps, the present study investigated a cohort of young adults who performed an inhibitory Go/NoGo task while undergoing functional magnetic resonance imaging (fMRI). In a well-powered design, we examined differences between BDs and LDs on a) behavioral indices of inhibitory control, b) inhibition-related patterns of neural activation, and c) associations between BOLD activation and alcohol-related self-reported variables, including alcohol consumption, drinking motives, and consequences. Building on previous evidence [56,57, 60,61], we hypothesized that BDs would exhibit greater BOLD activation during successfully inhibited NoGo trials relative to LDs in regions commonly associated with inhibitory control as a function of alcohol intake, but in the absence of observable behavioral deficits [61,84]. Our sample was adequately balanced across genders, permitting secondary analyses of potential gender-based differences in neural and behavioral outcomes. This approach fills in a gap in the existing literature and provides novel insights into how binge drinking may differentially impact inhibitory control networks in men and women during the early stages of alcohol misuse. Furthermore, using structural equation modeling, we examined whether the inhibition-related BOLD activation mediated the relationship between coping drinking motives and alcohol misuse outcomes for BD men and women.

## 2. Methods

### 2.1. Participants

The study participants included 83 right-handed, healthy young adults (44 women and 39 men, aged 22.1 ± 2.7 years). They reported no history of traumatic brain injury, seizures, neuropsychiatric disorders, vision or hearing problems, and no medication use at the time of the study. Participants reported no nicotine, cannabis, or illicit drug use at least one month before scanning. They were not seeking treatment for alcohol misuse and had not been previously enrolled in a treatment program. All participants complied with MRI safety conventions. The BD vs LD group assignment was based on a screening questionnaire and a follow-up interview that assessed the frequency, quantity, and rate of alcohol consumption. Binge episodes were defined as drinking occasions during which 5+/4+ (men/women) alcoholic drinks were consumed within two hours [1]. Participants who reported ≥ 5 binge episodes within the previous six months and at least one in the past month were assigned to the BD group. The LD group comprised participants who reported consuming alcohol regularly but at low levels. They engaged in ≤ 3 binge episodes in the previous six months and ≤ 1 in the past month. In the current sample, 27.2 % (*n* = 12) of LDs reported a single binge episode in the prior month, while all BDs engaged in binge drinking in the past month. BD and LD groups were equated across both genders on demographic variables, including age, gender, ethnicity, family history of alcohol use disorder, and general intellectual ability (Tables 1 and 2). The study’s procedures were approved by the Institutional Review Board at San Diego State University (HS-2019–0135) and complied with the ethical principles outlined in the Declaration of Helsinki. Written informed consent was obtained before administering any experimental procedures. Participants received monetary compensation for participating in the study.

### 2.2. Experimental protocol

All participants completed a battery of questionnaires assessing details of frequency, quantity, and pattern of alcohol consumption [85], presence of behaviors linked to alcohol use disorder [Alcohol Use Disorder Identification Test, AUDIT, [86]], alcohol consumption during the past thirty days [Time Line Follow Back, TLFB, [87]], the intensity of alcohol craving [The Penn Alcohol Craving Scale, PACS, [88]], motivations governing drinking behaviors [Drinking Motive Questionnaire Revised Short Form, DMQ-R SF, [89]], and the prevalence of negative consequences incurred through drinking [Brief Young Adult Consequences Questionnaire, B-YAACQ, [90]]. They also rated their depressive symptoms [Patient Health Questionnaire, PHQ-9, [91]], anxiety [Generalized Anxiety Disorder, GAD-7, [92]], degree of impulsive attributes associated with motor, non-planning, and attentional characteristics [Abbreviated Brief Impulsivity Scale, ABIS, [93]], the inclination for risk-taking and sensation seeking behaviors [Brief Sensation Seeking Scale, BSSS, [94]], perceived levels of stress [95], and overall sleep quality [Pittsburgh Sleep Quality Index, PSQI, [96]]. Cognitive abilities were assessed with the Wechsler Abbreviated Scale of Intelligence [WASI-II, [97]]. Family history of alcohol use disorder was evaluated with an abbreviated form of the Family History Assessment Module [FHAM, [98]]. A positive family history of alcohol use disorder was defined as having at least one immediate family member (father, siblings) and another immediate or extended family member (aunts, uncles, cousins) or 3+ extended relatives with a prior AUD diagnosis. Only participants with no maternal FH were included to avoid possible fetal alcohol exposure confounds. On the day of scanning, participants were screened with a 12-panel urine multidrug test (Discover, American Screening Corporation). Women were additionally screened for pregnancy, and all tests were negative.

### 2.3. Experimental paradigm

Inhibitory control was probed with a variant of the Go/NoGo task [26,52,61,84]. Participants were instructed to press a button with their right index finger to alternating X and Y letters (Go trials) and to withhold responding to repeating letters (e.g., X-X or Y-Y, NoGo trials). They were asked to respond as quickly as possible while maintaining accuracy and avoiding pressing the button prematurely before the stimulus presentation. All stimuli were presented in white font on a black background for 200 ms every 1300 ms (± 200 ms jittered in 50 ms increments) with Presentation v.19.0 software (Neurobehavioral Systems). A total of 700 trials were presented in four runs, comprising 560 (80 %) Go and 140 (20 %) NoGo trials, ensuring Go response dominance and engagement of inhibitory control by NoGo trials [47]. Optimized pseudorandomized stimulus sequences were generated with Optseq2 software [99] (https://surfer.nmr.mgh.harvard.edu/optseq/).

### 2.4. MRI data acquisition

Imaging data were acquired with a Siemens Prisma 3T scanner with a 32-channel head coil at the San Diego State University (SDSU) imaging center. Following a localizer scan, structural T1-weighted images were acquired using a 3D Magnetization-Prepared Rapid-acquisition Gradient Echo (MPRAGE) sequence with the following parameters: TR = 7.2 ms, TE = 3.01 ms, flip angle = 9°, TI = 900 ms, inversion repeat time = 2300 ms, bandwidth = 320 Hz/pix, FOV = 256 mm, matrix = 256 × 256, 176 axial slices, GRAPPA = 2, isotropic resolution of 1 mm. During task performance, functional blood-oxygen-level-dependent (BOLD) T2*-weighted images were acquired with an echo planar imaging (EPI) sequence of 45 axial oblique interleaved 3 mm slices oriented to encapsulate the entire brain. Functional images were acquired using the following parameters: TR = 1000 ms, TE = 32 ms, flip angle = 90°, FOV = 240 mm, matrix 80 × 80, in-plane resolution = 3 × 3 mm. We implemented steps to reduce motion artifacts while ensuring participant comfort during scanning.

### 2.5. Analysis of event-related fMRI-BOLD signal to correct NoGo vs Go trials

Analysis of Functional Neuro-Images (AFNI) software was used to examine functional imaging data [100,101]. To mitigate motion-related artifacts, all TRs with rotational and translational motion exceeding 0.3 mm and those with ≥ 20 % of voxels identified as outliers were removed. During deconvolution, six motion parameters were used to regress out motion along with a third-order polynomial to account for signal drift. Skull stripping and nonlinear alignment with the standardized MNI (TT_avg_152TI) template were accomplished with AFNI’s SSwarper program, and functional images were then coregistered to each participant’s SSwarper output. Smoothing was done with an 8 mm (FWHM) Gaussian kernel with voxels scaled to represent the percent signal change preceding deconvolution. A canonical hemodynamic response function (GAM) was used to model all trials. BOLD activation on correct NoGo trials was contrasted to that on Go trials, which served as the baseline. Error NoGo trials were excluded from the analysis. The AFNI program 3dDeconvolve was used to produce statistical maps for each participant with a residual maximum likelihood (REML) and generalized least squares (GLSQ) analysis method to identify voxels with significant differences from the Go baseline [102]. For second-level analysis, the AFNI function 3dttest++ was used to perform voxel-by-voxel two-sample *t*-tests comparing BD vs LD and gender-stratified subgroups across the entire brain volume for the activation elicited by correct NoGo trials. Cluster simulations were performed using 3dClustsim to ascertain the minimum cluster size required at the whole-brain level to detect group differences while controlling for family-wise error. Group difference was found at a cluster threshold of 43 contiguous voxels with a voxel-wise *p* = .0005, *q* = 0.01 (false discovery rate – FDR - corrected), and cluster-wise *p* = .05. Regions of interest (ROIs) were not defined *a priori* but were derived from clusters identified in the whole-brain analysis that met the specified significance threshold for the BD versus LD comparison [103,104]. Across all participants, beta coefficients representing percent signal change were extracted from each ROI for further analysis. Voxel-wise gender comparisons were conducted on the *NoGo* > *Go* contrast maps (*q* = 0.01, cluster-wise *p* = .05).

### 2.6. Analysis of behavioral data

Behavioral performance on the Go/NoGo task (accuracy and reaction time) was analyzed using mixed-model ANOVAs with trial type (Go, NoGo) as a within-subject factor with group and gender as between-subject factors. NoGo trials in which an erroneous response was recorded were removed from all analyses. No covariates were included in the behavioral or voxel-wise analysis. As shown in Tables 1 and 2, group differences in categorical variables were assessed with the *χ*^2^ test, while the non-parametric Mann-Whitney *U* test was used for all other group comparisons to account for potential violations of distribution normality. Correlations were carried out using non-parametric Spearman’s rank (rho) to investigate relationships between inhibition-related BOLD activation and self-reported variables related to alcohol intake and other behaviors and experiences, Go/NoGo performance, and personality/dispositional variables. The Benjamini-Hochberg procedure, based on a false discovery rate approach, was applied to correct for multiple correlations (FDR = 0.05) [105]. To examine the relationship between NoGo-specific LIFC activation, binge drinking, and coping motives as a function of gender, a moderated mediation analysis was conducted using the SPSS PROCESS Macro [106,107] with gender as a moderator and bias-corrected bootstrapping with 5000 iterations. Table 3 lists all estimated parameters along with 95 % confidence intervals.

## 3. Results

### 3.1. Participant characteristics

As shown in Table 1, BD and LD groups were equated based on age, gender, ethnicity, family history of alcohol use disorder, and general intellectual ability. As expected, consumption levels, alcohol-related behaviors, cravings, and negative consequences were higher in BDs relative to LDs. BDs reported higher motivations for drinking on the social and enhancement subscales. The two groups did not differ on measures of anxiety, depression, impulsivity, stress, or sleep quality. However, BDs did have higher scores on the boredom and disinhibition subscales of the BSSS. Group characteristics and statistical comparisons for men and women across both BD and LD groups are shown in Table 2. Not surprisingly, BD men (BDm) reported higher levels of habitual and binge drinking than BD women (BDw), as well as higher impulsivity. There were minimal gender differences in the LD group, with men (LDm) reporting higher scores on the thrill subscale of sensation seeking and enhancement drinking motives than women (LDw).

### 3.2. Task performance

**Fig. 1.**
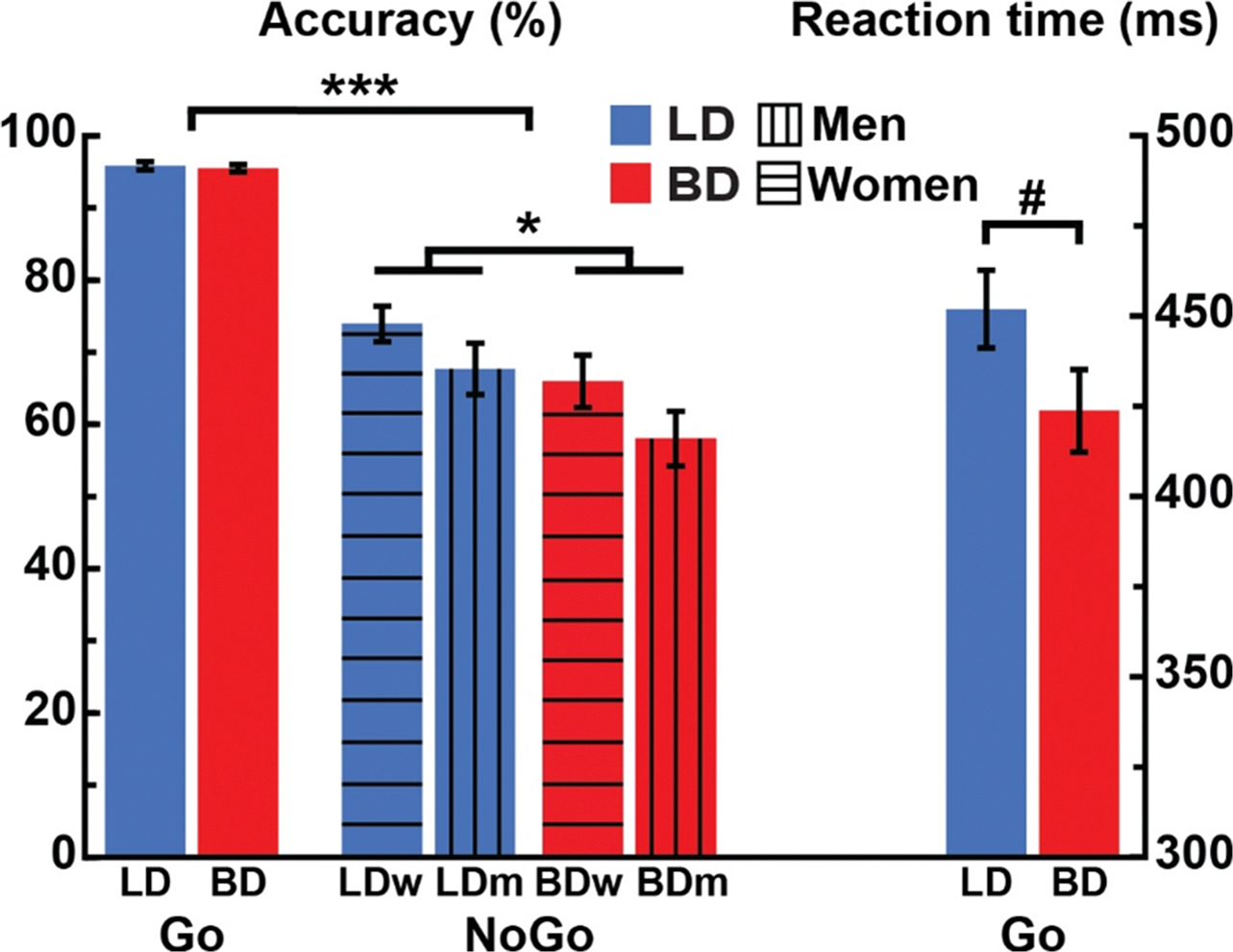
Go/NoGo task performance. a) Task accuracy for BD (binge drinking) and LD (light drinking) groups on Go trials overall. NoGo accuracy was separated by group and gender. BDs had lower NoGo accuracy relative to LDs. Women demonstrated better inhibitory control than men overall, as indicated by greater NoGo accuracy. b) BDs tended to respond faster than LDs on Go trials. *** *p* < .001, * *p* < .01, # *p* < .06

A mixed-design ANOVA indicated that BDs showed lower performance accuracy than LDs in the inhibitory (NoGo) condition, *F*_*1,79*_ = 6.78, *p* = .01, (Fig. 1). Women performed better than men overall on NoGo trials, *F*_*1,79*_ = 4.38, *p* = .04, but there was no group × gender interaction, *F*_*1,79*_ = 0.06, *p* = .80. None of the pairwise comparisons were significant, all *p’s* >0.07. As expected, a main effect of condition (*F*_*1,79*_ = 33.24, *p* < .001) was due to lower response accuracy on NoGo than on Go trials overall. There were no group or gender effects for Go trial accuracy, all *p’s* > 0.31.

BDs tended to have faster reaction times than LDs (*F*_*1,79*_ = 3.72, *p* = .06), but there were no gender differences (*F*_*1,79*_ = 1.81, *p* = .18), nor the group × gender interaction (*F*_*1,79*_ = 0.77, *p* = .38).

### 3.3. Inhibition-related BOLD activation

**Fig. 2.**
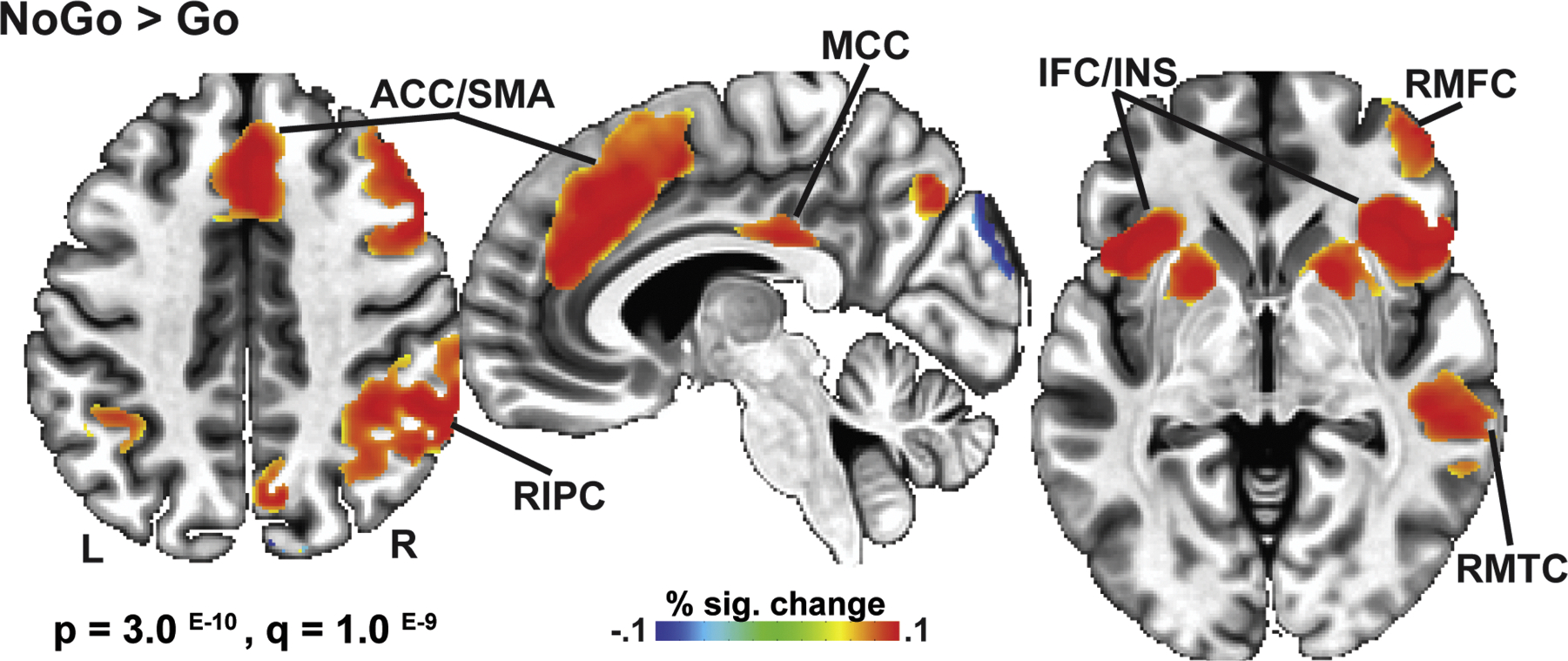
Overall BOLD activation pattern during successful inhibitory control. Collapsed across groups, greater BOLD activity was detected to NoGo trials relative to the Go trial baseline in distributed regions. Correct NoGo trials generated elevated BOLD activity in the anterior cingulate (ACC)/supplementary motor area (SMA), mid-cingulate (MCC), right middle frontal cortex (RMFC), bilateral inferior frontal cortices and adjoining insulae (IFC/INS), right inferior parietal cortex (RIPC), right middle temporal cortex (RMTC), and the basal ganglia.

The initial analysis included the entire sample (Fig. 2) to determine the overall BOLD activation pattern during the successful engagement of inhibitory control. BOLD contrast for NoGo > Go trials was thresholded at voxel-wise *p* = 3.0 ^E-10^, with false discovery rate (FDR) correction *q* = 1.0 ^E-9^. The NoGo-specific activation pattern included bilateral, but right-dominant, engagement of the prefrontal, parietal, and temporal cortices, along with the basal ganglia. Medially, NoGo activation was observed in the anterior cingulate/supplementary motor region, mid-cingulate, and precuneus.

**Fig. 3.**
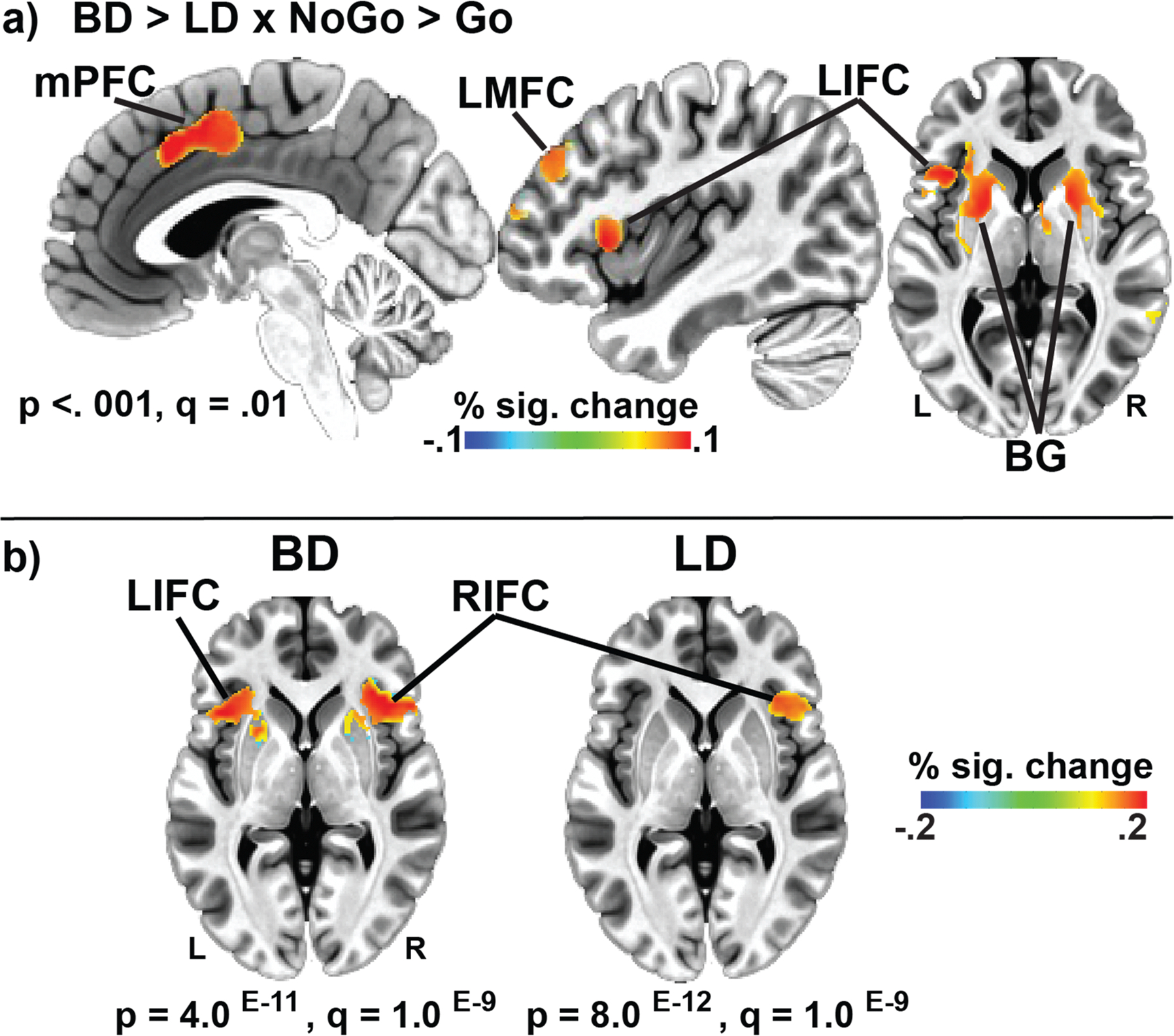
Group differences in BOLD activation to successful response inhibition (NoGo > Go). a) BDs showed greater BOLD activation than LDs in the medial prefrontal cortex (mPFC), left inferior frontal cortex (LIFC), left middle frontal cortex (LMFC), and basal ganglia bilaterally (BG). b) Prefrontal BOLD activation to the NoGo > Go contrast is shown separately for the BD and LD groups. While the right inferior frontal cortex (RIFC) is activated during NoGo inhibitory control in both groups, the LIFC is additionally engaged only in the BD group.

Inhibition-specific group differences (BD > LD × NoGo > Go BOLD contrast) revealed significant voxel-clusters in the medial prefrontal cortex (mPFC), left inferior (LIFC), and middle frontal cortices (LMFC), and basal ganglia bilaterally (Fig. 3a). Stronger BOLD activation was observed in the BD group, thresholded at p 〈 .001, with a FDR correction *q* = 0.01. BOLD activation values, representing percent signal change, were extracted from each cluster for all participants. Group comparisons of the NoGo 〉 Go contrast confirmed greater BOLD activation in BDs than LDs in the mPFC, F_1,79_ = 28.39, p 〈 .001, LIFC, F_1,79_ = 27.07, *p* < .001, and LMFC, F_1,79_ = 25.27, *p* < .001. Subcortically, the left, F_1,79_ = 30.66, *p* < .001, and right, F_1,79_ = 29.91, *p* < .001, basal ganglia were also more active in BDs relative to LDs. Fig. 3b shows activation patterns in BDs and LDs to the NoGo 〉 Go contrast. While the right IFC contributes to inhibitory control in both groups overall, the left IFC activation is prominent only in the BD group.

**Fig. 4.**
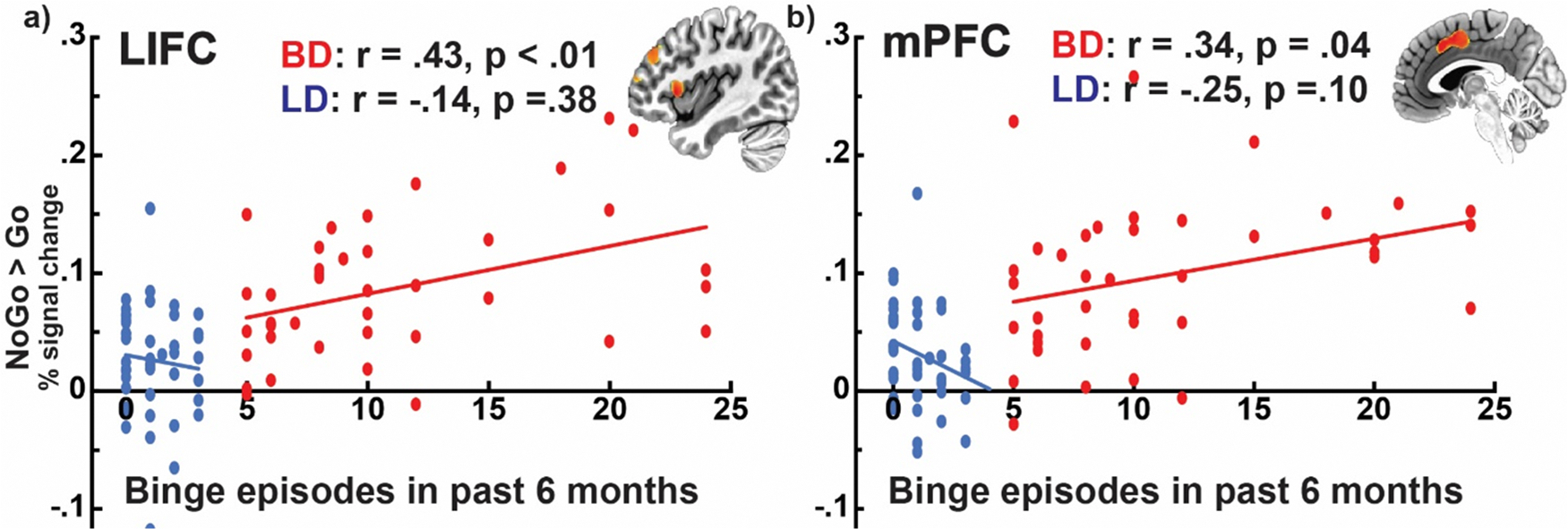
Prefrontal BOLD activation is associated with binge drinking. Scatterplots showing a positive correlation between inhibitory control (the NoGo > Go contrast) and binge drinking in BDs in the a) left inferior (IFC) and b) medial frontal (mPFC) cortices.

FDR-corrected correlations indicated that for the BD group, the number of binge drinking episodes in the previous six months correlated with greater NoGo-specific BOLD activation in the LIFC, *r* = 0.43, *p* < .01, and the left medial prefrontal cortex, *r* = 0.34, *p* = .04 (Fig. 4).

**Fig. 5.**
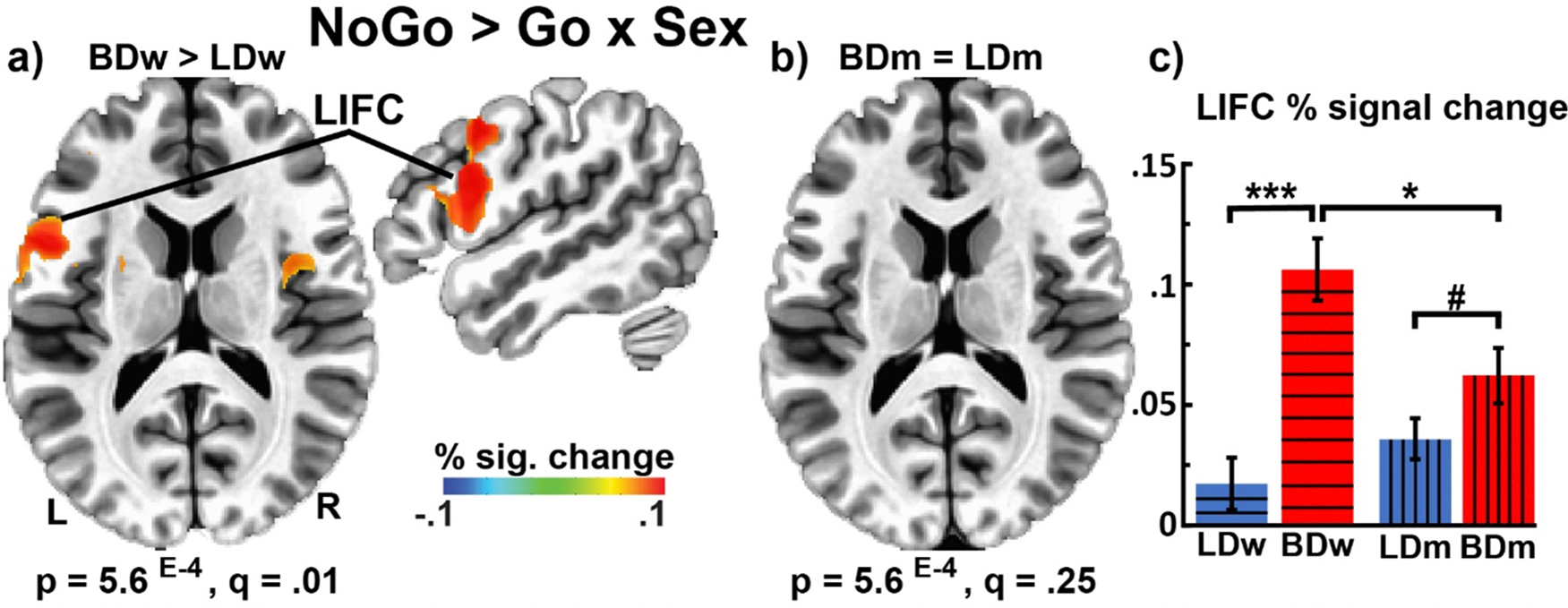
Gender-related differences in BOLD activation to NoGo trials. a) Binge-drinking women (BDw) showed greater activation than low-drinking women (LDw). The threshold was set at *p* = 5.6^E-4^, FDR *q* = 0.01, cluster-wise = 0.05. b) No reliable differences between BDm and LDm are observed at the same p-value threshold. c) Means ± SEM of BOLD activation within the LIFC for each gender and group separately. *** *p* < .001, * *p* < .05, # *p* < .06

As shown in Fig. 5, group comparisons for each gender revealed that BDw had greater LIFC activation than LDw, *F*_*1,42*_ = 28.01, *p* < .001. In contrast, the differences between BDm and LDm only trended towards significance, *F*_*1,79*_ = 3.64, *p* = .06. Gender comparisons for each group showed that BDw also had greater LIFC activation than BDm (*F*_*1,37*_ = 6.07, *p* = .02). Conversely, LDw did not differ from their LDm counterparts (*F*_*1,42*_ = 1.80, *p* = .19). These results suggest that binge drinking in women is associated with greater alterations in the neural correlates of inhibitory control compared with men.

A moderated mediation analysis was conducted to estimate the degree to which NoGo-specific LIFC activation mediates the impact of coping drinking motives on binge drinking levels as a function of gender. The overall indirect effect (*ab*) was significant only for women.

### 3.4. LIFC activation mediates the influence of coping motives on alcohol misuse in BDw

**Fig. 6.**
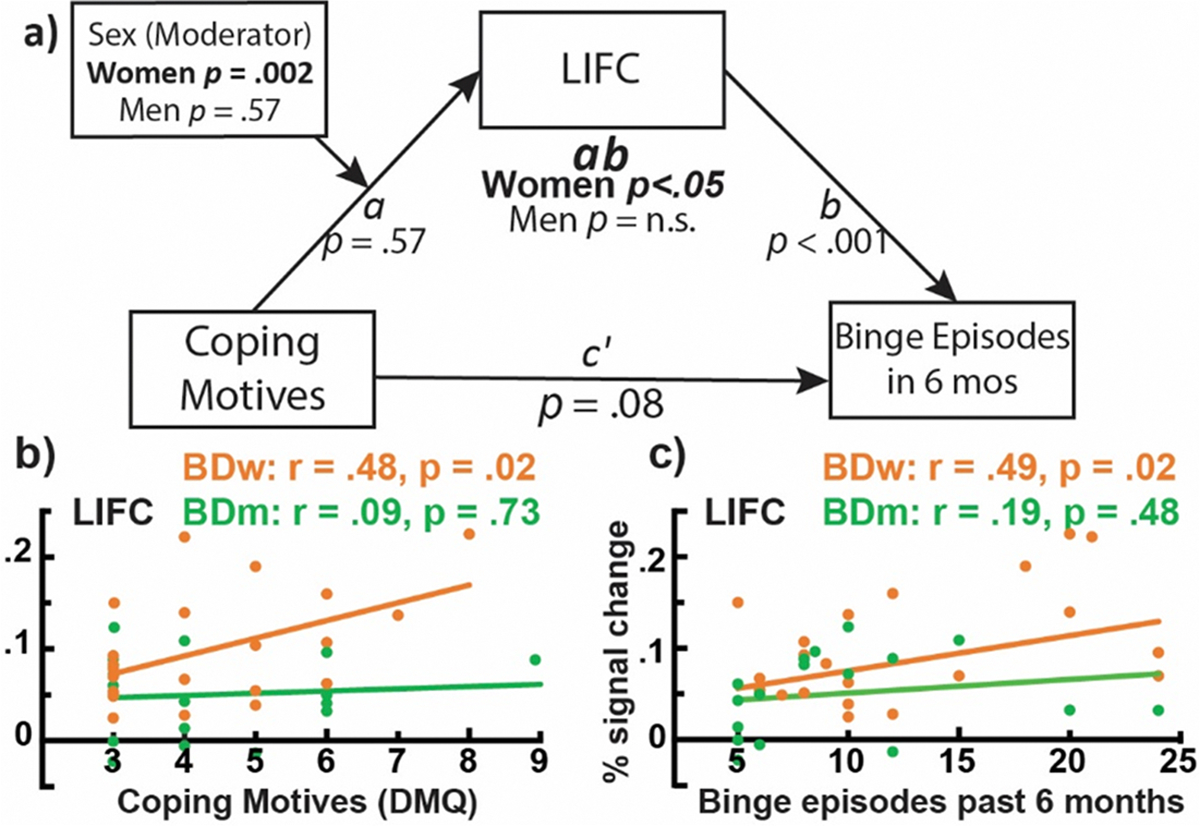
Moderated mediation analysis model with gender as a moderator. a) The impact of coping motives on binge drinking is indirectly mediated by LIFC activation to inhibitory control only for women. All model parameters are listed in Table 3. b) The scatterplot shows that motives to drink to cope with negative affect are correlated with LIFC activation to NoGo trials only for BD women. c) Similarly, NoGo-specific LIFC activation is associated with binge-level drinking only in BD women.

The observed gender-specific associations between LIFC activation and alcohol use were further explored with a moderated mediation analysis for men and women separately [107]. We tested whether LIFC activation mediated the effect of coping drinking motives on alcohol-use related behaviors. As shown in Fig. 6a and Table 3, the overall indirect effect (*ab*) was significant only for women, but not for men, indicating that LIFC activation mediated the impact of drinking to cope on higher alcohol misuse for women but not for men. Table 3 includes all the mediation model parameters. Relatedly, only BDw exhibited positive correlations between the LIFC activation and binge drinking, as reflected in the number of recent binge episodes (Fig. 6c), maximum number of drinks in the past six months, *r* = 0.48, *p* = 0.02 (Fig. 6c), and weekly consumption, *r* = 0.42, *p* = .05. These associations were not significant for BDm, *r* = 0.30, *p* = .25, and *r* = 0.22, *p* = .40, respectively. LIFC activation correlated with coping motives (Fig. 6b) for BDw, *r* = 0.48, *p* = .02, but not for BDm, *r* = 0.09, *p* = .73. Coping motives were related to higher anxiety for BDw only, *r* = 0.46, *p* = .03, BDm, *r* = 0.40, *p* = .11, as well as blackout occasions in the past six months, BDw *r* = 0.46, *p* = .03, BDm *r* = −0.05, *p* = .84, and weekly drinking, BDw *r* = 0.47, *p* < .03, BDm *r* = 0.17, *p* = .54.

## 4. Discussion

This study examined neurobehavioral indices of inhibitory control in young adult binge drinkers relative to those who typically consume alcohol in non-hazardous patterns. The primary group differences can be summarized as follows: a) BDs had lower NoGo accuracy and tended to respond faster than LDs, indicative of behavioral disinhibition (Fig. 1); b) inhibitory control elicited an expected overall BOLD activation pattern in the medial and lateral prefrontal, as well as posterior, and subcortical brain areas (Fig. 2); c) BDs showed greater BOLD response during NoGo trials in the LIFC, LMFC, mPFC, and BG bilaterally compared to LDs (Fig. 3); d) in the BD group, inhibitory BOLD signal in the LIFC and mPFC was positively associated with binge-level drinking over the past six months (Fig. 4). Investigation of gender-related effects revealed that e) overall, women outperformed men on inhibitory (NoGo) trials (Fig. 1); f) LIFC activation was higher in BDw than BDm during inhibitory control engagement on NoGo trials (Fig. 5); g) for women, LIFC activation mediated the impact of drinking to cope on alcohol misuse (Fig. 6a); Furthermore, LIFC activation was positively associated with drinking to cope and binge drinking for BDw only (Fig. 6b, c), consistent with previously reported gender differences in the predictive impact of negative emotionality on hazardous drinking.

Overall, the results are aligned with other evidence of elevated frontal engagement in BDs during an inhibitory control challenge in association with alcohol consumption levels. Our exploratory gender-based comparisons revealed that it is primarily BD women who show greater LIFC activation to inhibitory control which underlies maladaptive drinking to alleviate negative emotional states. The results support other evidence of women’s heightened sensitivity to alcohol misuse and increased risk of developing AUD.

### 4.1. Behavioral inhibition is deficient in BDs, potentially contributing to compulsive drinking

BDs made more commission errors than LDs by failing to inhibit responses on NoGo trials. In addition, they tended to respond faster on Go trials, indicating impaired inhibitory control (Fig. 1). This is a notable finding, as behavioral deficits in young BDs are not readily detected with behavioral measures, despite robust neural differences [56,58,61,84,108–116]. This disparity highlights a higher sensitivity of neuroimaging methods to emerging neurofunctional abnormalities in BDs. Additional factors, such as sample size, contribute to the likelihood of observing behavioral differences between BD and LD groups. For instance, previous studies employing this version of the Go/NoGo task in smaller samples reported comparable task performance but clear group differences in neural activity [61,84]. However, the present study was sufficiently powered to detect both behavioral inhibitory impairments and neural indices of inhibitory deficits in BDs. When noted, executive deficits in young BDs have been reported mainly in the inhibitory control domain [7,117]. Impaired inhibition predicts alcohol misuse [42–44], consistent with the importance of executive (dys)function as an essential neurofunctional domain within the ANA framework [36,41]. Conversely, inhibitory control may play a protective role in reducing compulsive alcohol consumption and the risk of alcohol-related negative consequences [118]. Indeed, people with AUD show impaired inhibitory control [37,40,119–121], while the evidence in nondependent drinkers is mixed [122,123]. Women performed the task more accurately than men (Fig. 1), consistent with other evidence of more effective behavioral inhibition in women, specifically on Go/NoGo tasks [75,124]. Furthermore, neuroimaging studies have reported greater BOLD activation during successful NoGo inhibition in women than in men [125].

### 4.2. Binge drinking is associated with greater BOLD activation, suggesting compensatory engagement

Across all participants (Fig. 2), NoGo trials evoked a BOLD activation pattern consistent with extensive evidence demonstrating that inhibitory control is governed by a right-dominant network with major contributions from the prefrontal cortex [26,45,48,50,51,126]. Furthermore, connectivity studies have underlined the importance of the fronto-striatal pathways for inhibitory functions [127]. Interactive signaling between the medial and lateral prefrontal cortices and BG may provide the basis of inhibitory control, allowing for NoGo detection and stopping of prepotent motor responses [25,45,128–131].

In the current study, successful NoGo inhibition elicited greater BOLD activation in BDs relative to LDs in the left-lateralized prefrontal cortices, medial prefrontal area, and BG (Fig. 3a). It has been established that the mPFC, comprising the anterior cingulate cortex (ACC) and the supplementary motor area (SMA), is implicated in monitoring for potential response conflict, behavior optimization, and integration with motivational and strategic goals [132,133]. Engagement of the mPFC is also associated with the selection, preparation, inhibition, and execution of motor responses [27,134,135]. Inhibitory NoGo trials require moment-to-moment updating of stimulus-response representation to switch between prepotent motor responses to Go trials and the controlled NoGo response suppression effectively [50]. Furthermore, its functional connectivity with the lateral frontal cortex [136] positions the mPFC as an inhibitory control hub, confirmed by inhibition deficits in patients with insults to this region [137,138]. Meta-analyses have shown that the mPFC is one of the most commonly activated regions during inhibitory tasks [48–50]. The right inferior frontal cortex (IFC) has been characterized by extensive evidence as the dominant neural substrate involved in inhibitory processes [26,45,48,52], including attentional aspects [54,55,139,140]. The question of the left vs. right IFC inhibitory dominance has been vigorously debated. Although right-dominant, inhibition-related overall BOLD activation is bilateral in the present study (Fig. 2), which is aligned with other imaging evidence [45,48–51,55,140,141]. However, BDs exhibited greater activation of the *left* IFC and MFC, and bilateral mPFC and BG during NoGo inhibitory probes (Fig. 3b). While the right IFC was activated in both groups, BDs additionally activated the left IFC to a greater degree, presumably to bolster the inhibitory contributions of the right IFC (Fig. 3). LIFC and mPFC activation was associated with the number of binge episodes in the past six months (Fig. 4), suggesting that individuals with greater alcohol misuse may engage compensatory neural recruitment to maintain inhibitory control.

Research on inhibitory control deficits in BDs is scarce. However, the majority of the evidence suggests greater BOLD activation in BDs than in LDs on NoGo trials in frontal and other areas [56,57,59,142]; but see Hu et al. [59]. Greater BOLD activation has also been observed in youth after transitioning to heavy drinking relative to those who remained alcohol naïve [143], suggesting that excessive alcohol consumption may disrupt the refinement of typically developing neural pathways, necessitating compensatory adjustments. Aligned with such observations, higher levels of BOLD activation in BDs have additionally been reported in studies probing cognitive control [60,144], error processing [61], and working memory [62,145]. Similarly, abstinent people with AUD show greater activation in bilateral [146] and medial prefrontal regions [147, 148] in response to inhibitory NoGo demands, as well as in other cognitive domains, including error processing [149], response interference [150], and working memory [151–153]. Taken together, greater BOLD activation may reflect compensatory engagement of neural resources during demanding tasks to maintain normative functioning [61, 154]. In contrast, lower BOLD activation is commonly observed in people with current AUD [155,156] who manifest neurofunctional deficits resulting from diminished neural reserve while drinking excessively [157].

### 4.3. Inhibitory control activates the left IFC in BD women in association with binge drinking, coping motives, and negative emotionality

Though exploratory, our findings on gender differences provide insight into divergent trajectories of prefrontal top-down inhibitory control over behavior as a function of binge drinking. More specifically, activation of the LIFC was observed only in BDs (Fig. 3), which was greater in BDw than in BDm during inhibitory control (Fig. 5). LIFC is part of the salience network, which is sensitive to both cognitive and emotional aspects [158]. Increased LIFC activation has been reported in response to stressful stimuli [159] and alcohol cues in women with AUD, which predicted higher drinking levels [160]. In the present study, the fast-paced, inhibitory version of the Go/NoGo task was likely aversive and stressful, eliciting negative affect in BD women. Task difficulty ratings correlated with anxiety for BDw (*r* = 0.45, *p* = .05) but not BDm (*r* = −0.41, *p* = 0.21). Importantly, the LIFC activation correlated with binge and habitual alcohol consumption, as well as with drinking to alleviate negative emotions (i.e., coping motives) for BDw only (Fig. 6). Moreover, inhibition-related LIFC activation mediated the impact of coping motives on binge drinking for women but not men (Fig. 6). Coping motives correlated with alcohol consumption and anxiety for BDw, which is suggestive of emotion dysregulation. This aligns with a broader picture of the high prevalence of negative emotionality among people with AUD, especially women [161–165]. Women are more likely to drink to self-medicate and to cope with adverse life events than men [166], particularly during young adulthood [167,168]. Thus, increased LIFC activation in BDw may indicate an overtaxed inhibitory control network as greater emotional reactivity engenders increased coping motives, resulting in elevated drinking levels [169,170]. This is consistent with a compensatory account and reappraisal, as prefrontal areas modulate limbic contributions [171], which is reflected in the engagement of the LIFC during reappraisal and emotion down-regulation [172–174]. Impaired emotion regulation has been implicated as a transdiagnostic dimension contributing to psychopathological conditions, including addiction [175]. The role of coping motives in LIFC engagement and in predicting women’s binge drinking is aligned with the importance of executive (dys)function and negative emotionality dimensions in the ANA framework with relevance to AUD diagnosis and treatment [36]. This pattern may reflect a maladaptive coping strategy resulting in women’s higher susceptibility to the adverse effects of alcohol and their faster (“telescoped”) progression toward AUD [159]. Although previous research has reported gender differences in drinking motives [176,177] several factors may account for the absence of gender differences in the current sample. First, men and women in this study reported comparable drinking frequency and quantity, which may have reduced variance attributable to gender. Importantly, the sample of young adults is drawn from similar social contexts, where opportunities and norms for alcohol use are comparable for young men and women. Furthermore, gender gaps in drinking behaviors and related motives have narrowed over time, reflecting broader cultural shifts toward more equivalent patterns of alcohol use [178, 179]. Finally, a limited range in motive scores may have constrained the detection of subtle gender-related differences. Together, these factors suggest that the motivational underpinnings of drinking in young adulthood may be more similar for men and women than previously observed in other samples.

Overall, the study provides evidence of altered prefrontal and striatal activation in young adults as a function of alcohol misuse. These results align with the concept of compensatory neural engagement when challenged with demanding task requirements. Furthermore, the additional analyses exploring gender-stratified subgroups suggest that alcohol-related neural alterations may contribute to the risk of binge drinking, higher levels of anxiety, and emotion dysregulation differently in men and women. Given the interactive impacts of the executive and affective ANA domains, additional well-powered studies of inhibitory control focused on gender-specific differences are needed to replicate these results, provide further evidence of its involvement in alcohol misuse, and inform the development of interventions in young emerging adults.

Findings from the current study should be considered in light of several limitations. The question of whether the observed effects are a product of pre-existing traits or manifest as a function of heavy drinking cannot be addressed due to the cross-sectional design of the study. Likewise, the mediation analysis suggested that inhibition-related LIFC activation mediated the impact of coping motives on higher rates of binge drinking in women. However, this was cross-sectional rather than a longitudinal design, lacking temporal sequencing to establish implied causality. Inclusion of LDs who have engaged in limited binge drinking may have reduced the otherwise clear contrast between BD and LD groups in terms of their recent drinking profiles. However, this strengthens the observed group differences in brain activity and enhances the study’s representational validity. Exclusion of recent nicotine and cannabis use may impact its generalizability to the broader young adult population, among whom polysubstance use is common. On the other hand, this restriction strengthens internal validity by allowing the observed results to be more directly attributed to alcohol misuse rather than to comorbid substance use.

In conclusion, BDs showed enhanced BOLD activation in the frontal and subcortical areas during successfully inhibited NoGo trials compared to LDs. Left-dominant prefrontal activation to NoGos correlated with binge drinking levels in BDs. In conjunction with inhibitory task performance deficits, this evidence is suggestive of impaired inhibitory control in young emerging adults who regularly engage in binge drinking. Notably, the observed group differences primarily pertained to BD women, who showed greater NoGo-related LIFC activation than BD men. Moreover, LIFC activation in BD women was found to mediate the association between drinking to cope with negative emotionality and hazardous drinking. These findings are consistent with other evidence of greater women’s vulnerability to harmful effects of alcohol and the importance of emotional dysregulation for alcohol misuse. They elucidate the need for further research on the interactions between neurobehavioral deficits, binge drinking, and coping mechanisms in men and women, in the service of developing targeted interventions to mitigate these complex gender-dependent impacts.

## Figures and Tables

**Table 1:** Participant characteristics for BD and LD groups.Participants.

Participant	Binge (*n* = 39)	Light (*n* = 44)	*U*/χ^2^	p-value

Age	21.6(±2.6)	22.5(±2.7)	703	.15
% Women ^a^	56	50	.47	.49
Ethnicity (white, non-Hispanic) ^a^	54 %	52 %	.31	.96
Family History of Alcohol Use Disorder ^a^	15 %	16 %	.004	.95
WASI-II % rank (FSIQ-2)	70.8(±17.4)	72.4(±19.3)	793	.55
**In the past six months**				
Drinking days per week	2.0(±1.0)	1.1(±.88)	423	**<0.001**
Drinks per occasion	4.4(±2.7)	1.8(±1.5)	267	**<0.001**
Drinks consumed per week	7.6(±4.4)	2.6(±2.6)	218	**<0.001**
Binge episodes	11.1(±6.0)	1.1(±1.2)	.000	**<0.001**
Alcohol-related blackouts	2.2(±3.1)	.33(±.74)	432	**<0.001**
Max no of drinks in 24h	12.3(±5.6)	5.3(±2.9)	197	**<0.001**
Binge episodes in the past month	3.0(±1.7)	.27(±.45)	48	**<0.001**
Age of onset of alcohol use	16.3(±2.3)	17.3(±1.7)	595	**.01**
Alc. Use Disorder Ident. Test (AUDIT)	8.9(±3.6)	4.6(±2.6)	277	**<0.001**
Alcohol cravings (PACS)	4.6(±4.0)	2.5(±2.7)	507	**.002**
Consequences of alcohol use (B-YAACQ)	5.3(±4.0)	2.5(±3.5)	396	**<0.001**
Motivations for drinking (DMQ-R)				
Social	7.5(±1.3)	6.2(±1.8)	472	**<0.001**
Coping	4.3(±1.6)	3.8(±1.1)	696	.16
Conformity	4.3(±1.5)	4.0(±1.3)	763	.47
Enhancement	6.5(±1.1)	5.3(±1.7)	444	**<0.001**
Anxiety (GAD-7)	4.7(±4.0)	4.7(±5.1)	783	.62
Depression (PHQ-9)	3.9(±3.8)	3.9(±4.4)	806	.78
Impulsivity (ABIS)				
Motor	7.9(±1.9)	7.2(±2.0)	653	.08
Attention	8.9(±1.7)	8.8(±2.6)	767	.52
Non-planning	7.5(±2.8)	6.8(±2.4)	746	.40
Sensation Seeking (BSSS)				
Experience	7.6(±1.6)	7.0(1.8)	723	.29
Boredom	7.3(±1.3)	6.6(1.6)	622	**.04**
Thrill	6.2(±2.2)	6.0(±2.0)	815	.84
Disinhibition	6.6(±1.5)	5.5(±1.8)	564	**.01**
Perceived stress (PSS)	20.2(±3.6)	19.8(±5.3)	834	.99
Sleep quality (PSQI)	4.6(±2.9)	4.2(±2.4)	809	.80

a Tested with Chi-Square; all other comparisons were performed using a non-parametric Mann-Whitney *U* test.

Significant p-values are marked in boldface font. WASI-II: Wechsler Abbreviated Scale of Intelligence; AUDIT: Alcohol Use Disorder Identification Test; PACS: Penn Alcohol Craving Scale; DSM-V Diagnostic Criteria for Alcohol Use Disorder; B-YAACQ: Brief Young Adult Alcohol Consequences Questionnaire; DMQ-R: Drinking Motivations Questionnaire-Revised; GAD-7: Generalized Anxiety Disorder; PHQ-9: Patient Health Questionnaire; ABIS: Abbreviated Impulsiveness Scale; BSSS: Brief Sensation Seeking Scale; PSS: Perceived Stress Scale; PSQI: Pittsburgh Sleep Quality Index.

**Table 2:** Gender-based participant characteristics for BD and LD groups.

Participants	BDm (*n* = 17)	BDw (*n* = 22)	LDm (*n* = 22)	LDw (*n* = 22)	BDm v BDw	LDm v LDw	BDm v LDm	BDw v LDw

Age	22.2(±3.1)	21.2(±2.3)	23.2(±3.1)	21.8(±2.2)				
Ethnicity ^a^	53 %	55 %	55 %	52 %				
Family history ^a^	12 %	18 %	9 %	22 %				
WASI-II % rank	69.1(±20.1)	72.1(±15.3)	69.3(±21.7)	75.5(±17.1)				
**Past six months**								
Drinking days/wk	2.0(±1.2)	2.0(±.88)	1.1(±.91)	1.1(±.79)				***
Drinks/occasion	5.7(±3.5)	3.5(±1.4)	1.9(±1.7)	1.8(±1.3)	*		***	***
Drinks per week	8.7(±5.2)	6.8(±3.5)	2.5(±2.3)	2.6(±2.8)			***	***
Binge episodes	9.7(±5.6)	12.2(±6.2)	1.1(±1.d	1.0(±1.1)			***	***
Blackouts	2.2(±3.8)	2.2(±2.6)	.27(±.70)	.27(±.55)				***
Max drinks in 24h	14.6(±6.3)	10.5(±4.3)	5.8(±2.9)	4.7(±3.0)	*		***	***
Binge ep past mo	2.8(±1.7)	3.2(±1.7)	.27(±.45)	.27(±.45)			***	***
Alcohol onset	16.4(±3.2)	16.3(±1.4)	17.8(±1.6)	16.8(±1.8)			*	
AUDIT	9.8(±3.3)	8.2(±3.8)	4.3(±2.3)	4.8(±3.0)			***	**
PACS	5.6(±5.1)	3.9(±2.9)	2.5(±2.5)	2.3(±3.0)				*
B-YAACQ	5.1(±3.3)	5.4(±4.5)	2.7(±3.7)	2.3(±3.4)			**	**
DMQ-R								
Social	7.4(±1.3)	7.5(±1.3)	6.3(±2.0)	6.0(±1.6)				**
Coping	4.3(±1.7)	4.4(±1.5)	4.0(±1.3)	3.8(±1.1)				
Conformity	4.4(±1.9)	4.1(±1.1)	3.8(±1.3)	4.2(±1.3)				
Enhancement	6.8(±1.2)	6.3(±1.0)	5.8(±1.8)	4.6(±1.4)		*		***
GAD-7	4.6(±4.0)	4.9(±4.0)	3.9(±5.2)	5.7(±4.0)				
PHQ-9	4.3(±4.4)	3.7(±3.4)	3.7(±4.6)	4.1(±5.1)				
ABIS								
Motor	8.5(±1.7)	7.5(±2.0)	7.4(±1.8)	7.1(±2.2)				
Attention	9.3(±1.6)	8.7(±1.8)	9.1(±2.6)	8.5(±2.6)				
Non-planning	8.9(±3.6)	6.5(±1.4)	7.2(±2.5)	6.6(±2.3)	*			
BSSS								
Experience	7.9(±1.5)	7.3(±1.6)	7.3(±2.0)	7.0(±1.5)				
Boredom	7.5(±1.4)	7.2(±1.3)	6.5(±1.9)	6.7(±1.4)				
Thrill	6.9(±2.3)	5.6(±2.1)	6.7(±1.9)	5.3(±1.9)		*		
Disinhibition	7.5(±1.4)	6.0(±1.3)	6.0(±1.9)	5.0(±1.7)	**		*	
PSS	19.3(±3.9)	20.8(±3.3)	19.3(±4.7)	20.3(±6.0)				
PSQI	4.3(±2.5)	4.9(±3.2)	3.8(±2.1)	4.7(±2.5)				

a Tested with a chi-square test; all other comparisons were performed using a non-parametric Mann-Whitney *U* test. All acronyms are described in the legend for Table 1.

Significant differences are marked with * *p* ≤ 0.05, ** *p* ≤ 0.01, *** *p* ≤ 0.001.

**Table 3:** Mediation results.

Parameter	Est.	*p*	*95 % C.I.*

a	−0.0036	.571	[−0.0160 −0.0089]
Gender	−0.0854	**.032**	**[−0.1633 −0.0075]**
a x Gender	.0241	**.009**	**[.0061 0.0421]**
Men	−0.0036	.571	[−0.0160 0.0089]
Women	.0205	**.002**	**[.0076 0.0335]**
b	55.56	**<0.001**	**[34.88 76.24]**
c’	.80	.076	[−0.09 1.69]
Men ab	−0.20		[−0.97 0.32]
Women ab	1.1**4**		**[.09 2.04]**

## Data Availability

The data that support the findings of this study are available from the corresponding author upon reasonable request.
